# Review of “*Molecular Biology of Kinetoplastid Parasites*” edited by Hemanta K. Majumder

**DOI:** 10.1186/s13071-018-2978-2

**Published:** 2018-07-11

**Authors:** Helen P. Price

**Affiliations:** Centre for Applied Entomology and Parasitology, School of Life Sciences, Keele University, Newcastle-under-Lyme, Staffordshire ST5 5BG UK

## Abstract

Majumder HK, Editor.

*Molecular Biology of Kinetoplastid Parasites.*

Poole, UK: Caister Academic Press; 2018, 246 pages.

ISBN 978-1-910190-71-5.

## Review

It has been 12 years since the genomes of the ‘TriTryps’ (*Leishmania major*, *Trypanosoma brucei* and *Trypanosoma cruzi*) were first published. Since that time, the kinetoplastid research community have come a long way in understanding the unique biology of these parasites. Tools such as CRISPR/Cas9, RNA interference library screening and next generation sequencing are now allowing investigations on a scale that was previously impossible. We have been able to make great progress in exploring topics such as the mechanisms of drug resistance and understanding how specific populations have evolved. It is appropriate that a new book is available to help explain new developments in the molecular biology of the kinetoplastids in the context of existing knowledge in this field.

This book is divided into 13 chapters, each of which is written as a separate article. For future editions, the book may benefit from a short introductory chapter which would allow the introductory paragraphs to be removed from each chapter, thus reducing some repetition. The dominant focus of the book is on *Leishmania*, reflecting the expertise of the authors. While there is considerable depth on molecular biology in some sections, other parts of the book are more immunology-focused.

Chapter 1 is written as an experimental paper and describes the use of bioinformatics to identify chromatin remodelling proteins in *L. infantum*. This is a rich source of information for researchers with an interest in this topic, although a more extensive section on epigenetic regulators as druggable targets may also be useful. Chapters 2–6 review a range of topics in immunology and the complex interactions between the *Leishmania* parasite and the host. The chapter on the response of B-lymphocytes (Chapter 3) is particularly detailed and gives a comprehensive description of the development of B-cell populations and the roles of regulatory B-cells in addition to immunopathology in *Leishmania* infections. Chapter 7 reviews the role of host ceramide, a component of sphingomyelins, in the establishment of visceral leishmaniasis. Chapters 8 and 9 focus respectively, on the roles of haem proteins during the life-cycle of *Leishmania*; and key changes in the expression of structural proteins, virulence factors and stress related proteins during the differentiation of *Leishmania* into host-infective forms. Chapters 10–12 have a wider focus on kinetoplastids, covering topoisomerases, host-parasite interactions, extracellular matrix interacting proteins. The final chapter of the book is the most translational of the articles and discusses the effects of phospholipid analogues on trypanosomatids.

There is some variation in the use of figures in the book. While many of the chapters are well illustrated, some images need to be reproduced in larger format and at higher resolution in order to view them easily; this could be considered for future editions.

In conclusion, this book provides a detailed account of a number of essential processes in kinetoplastids, covering *Leishmania* in much greater depth than other parasites. I would therefore recommend this book to researchers with an interest on leishmaniasis, particularly those who wish to expand their broad knowledge of the parasite and its host interactions. In future editions it may be useful to include more on the molecular tools that are allowing us to explore the biology of kinetoplastids, and to describe new findings with importance to the field such as the identification of hybrids and the remarkable ability of the *Leishmania* species to undergo aneuploidy.

